# Subjective Information and Survival in a Simulated Biological System

**DOI:** 10.3390/e24050639

**Published:** 2022-05-02

**Authors:** Tyler S. Barker, Massimiliano Pierobon, Peter J. Thomas

**Affiliations:** 1 School of Computing, College of Engineering, University of Nebraska-Lincoln, Lincoln, NE 68588, USA; tyler.barker@huskers.unl.edu; 2 Department of Mathematics, Applied Mathematics and Statistics, Case Western Reserve University, Cleveland, OH 44106, USA; pjthomas@case.edu

**Keywords:** mutual information, biology, foraging, chemotaxis, growth rate, subjective information

## Abstract

Information transmission and storage have gained traction as unifying concepts to characterize biological systems and their chances of survival and evolution at multiple scales. Despite the potential for an information-based mathematical framework to offer new insights into life processes and ways to interact with and control them, the main legacy is that of Shannon’s, where a purely syntactic characterization of information scores systems on the basis of their maximum information efficiency. The latter metrics seem not entirely suitable for biological systems, where transmission and storage of different pieces of information (carrying different semantics) can result in different chances of survival. Based on an abstract mathematical model able to capture the parameters and behaviors of a population of single-celled organisms whose survival is correlated to information retrieval from the environment, this paper explores the aforementioned disconnect between classical information theory and biology. In this paper, we present a model, specified as a computational state machine, which is then utilized in a simulation framework constructed specifically to reveal emergence of a “subjective information”, i.e., trade-off between a living system’s capability to maximize the acquisition of information from the environment, and the maximization of its growth and survival over time. Simulations clearly show that a strategy that maximizes information efficiency results in a lower growth rate with respect to the strategy that gains less information but contains a higher meaning for survival.

## 1. Introduction

Information processing and its correlation with survival and evolution in living organisms have been proposed through the years as unifying concepts to study life across different scales [1]. The transmission and storage of information are indeed at the basis of very diverse life processes, ranging from the processing of information via molecular interactions within a cell to complex codes in inter-organismal communication, and the consequent emergence of long-term evolutionary patterns [2]. Interestingly, while the development of a suitable mathematical framework to study and quantify information in these contexts could revolutionize the way we characterize, analyze, and interact with biological systems, there is little agreement on the mathematical formalism to be applied [3], mostly based upon Shannon’s proposed framework.

In its inception, the restriction of Shannon’s mathematical theory of communication [4,5] to purely syntactic considerations, where the meaning of information is considered irrelevant to the engineering problem, was a conceptual *sine qua non* for its success and applicability within the emerging fields of electronics and telecommunications. In particular, the concept of information entropy as a universal metric to quantify information, or its absence, i.e., noise, and the definition of the capacity of a communication channel as the theoretical maximum amount of information, or Mutual Information (MI) that can be unequivocally propagated between two points in space (transmission) or time (storage), have been the fundamental underpinnings in the study of mathematical algorithms where information can be represented and received with maximum efficiency.

Despite attempts to apply the aforementioned concepts in biology, from neuroscience to biochemistry [6], and data analytics for bioinformatics [7], even abstracting biological systems as communication channels [8,9,10,11], the syntactic nature of information theory provides an obstacle to its application to living systems. Intuitively, within biological systems, some messages are “more important” than others. There have been attempts to provide a quantitative basis for this idea [12,13,14] but a general framework remains lacking.

Here, we develop a simple but rigorous model that illustrates the notion of “**subjective information**”, defined as the particular information that an individual organism focuses on, out of all the potentially *useful* information available to the organism. We show that, under certain conditions, maximizing the expected cell division rate (or fitness) requires maximizing information about a sequence of selected subsets of an input signal’s components in a way that deliberately discards a larger quantity of less useful information in favor of a smaller quantity of more useful information. The work presented in the following builds on preliminary results included in a previous conference publication [15]. In this extended version, we formulate a mathematical model of the biological system alongside the computational model, and we include the modeling and simulation of death and division processes, and introduce a new metric to measure cell population growth to better capture the correlation between information and organism survival. Comprehensive results presented in Section 5 now enable us to study the effects of cell stress on survival and its correlation to subjective information, and to propose a simple metric to quantify the emergence of subjective information in a population.

Other models and frameworks to study information in the context of agents receiving information to maximize their fitness have been proposed, either in the context of biology or in more general terms. In particular, a trade-off between a reward or fitness purpose (value-to-go) experienced by an organism versus how much informational “bandwidth” can be afforded (information-to-go) for that purpose is analyzed in [14] with a Markov Decision Process (MDP) formalism. These authors find a trade-off, under the constraint of a cost for information acquisition, by applying a reinforcement learning optimization to an MDP model of the organism. In [16], a thermodynamic-based framework (instead of purely information-theoretic) is employed to study decision-making processes where a constraint on the information processing resources needed to achieve optimal decision for maximum reward is considered. Under this framework, a trade-off between reward and information processing cost is found as a function of this constraint through the theory of free-energy differences and thermodynamic energy potentials. When processing costs are ignored, the maximum utility is reached with maximum use of information. In [17], a clear distinction about relevant and irrelevant information for an organism is made by proposing a metric to account for the fitness value of information, or *Gould–Kelly* information [18], i.e., the increase in the organism’s fitness resulting from a cue conveying (limited) information about the environment. In addition, an upper bound limit to the fitness value is found as the mutual information between this cue and the environment, which can be easily interpreted as the performance of the communication channel between environment and organism. In a more recent publication [19], the fitness value of information and its upper bound are generalized for situations where the environment can vary fitness related parameters in time and/or space, possibly characterized by gradients, where the value of an informative cue to the organism becomes a function of the probabilistic distribution of these parameters. A definition of “semantic information” is proposed in [20] as the minimum amount of (relevant) information for an organism to ensure maximum viability (fitness). This quantity is found by gradually degrading a single information channel between the environment and the organism through a “coarse-graining” function until a decrease in the organism fitness is observed. It is here assumed that providing the organism with more information than the semantic information does not contribute to its fitness.

All these previous contributions, amongst other results, seem to conclude that, for a biological organism (or a model/generalization thereof), more “relevant” information is always better, with its cost of acquisition or processing being the constraint, i.e., there is always a trade-off between utility/reward and work to be done. In contrast, our contribution shifts the focus from constraints on information cost, to constraints in the capability of an individual organism to opportunistically manipulate the information channel between the environment and itself. As we demonstrate through our model, where we consider two essential metabolic sources for growth and survival corresponding to competing space-varying chemotactic information cues from the environment, the variability of this information channel across the organism population has the two-fold effects of increasing the overall fitness while also decreasing the average information necessary to achieve this fitness.

The rest of the paper is organized as follows: in Section 2, we introduce a mathematical abstraction and consequent mathematical and computational models of a living system exhibiting the aforementioned characteristics, based on an organism that requires two essential molecular species, and information on their distribution, to survive and grow. In Section 3, we define two metrics to quantify information and survival from the output of simulations based on the model organism. In Section 4, we introduce and discuss how the simulation framework is implemented from the computational model. In Section 5, we present numerical results obtained from our simulations and the subsequent estimation of the metrics, while, in Section 6, we discuss our findings, finally proposing a simple metric to quantify the emergence of subjective information in the simulated living system. Finally, we conclude the paper in Section 7.

## 2. Two-Resource Foraging Model

In this paper, we consider an abstract model of an organism that requires two essential metabolic substrates to survive and grow, and whose behavior is simple but essential for appreciating the concept of “subjective information” and its correlation with survival. In this model, the two substrates have different spatial distributions, and the organism detects their local concentrations and gradients through a noisy receptor-binding process [21,22], which in turn informs its chemotaxis [23]. This model is then used to compare the organism survival (in terms of population growth rates [18,24]) upon adoption of strategies based either on maximizing information on the two essential substrates [25] or, alternatively, reducing this information by focusing on what is more important for survival [12], which corresponds to the emergence of a “subjective information”.

### 2.1. Mathematical Model

**Figure 1:**
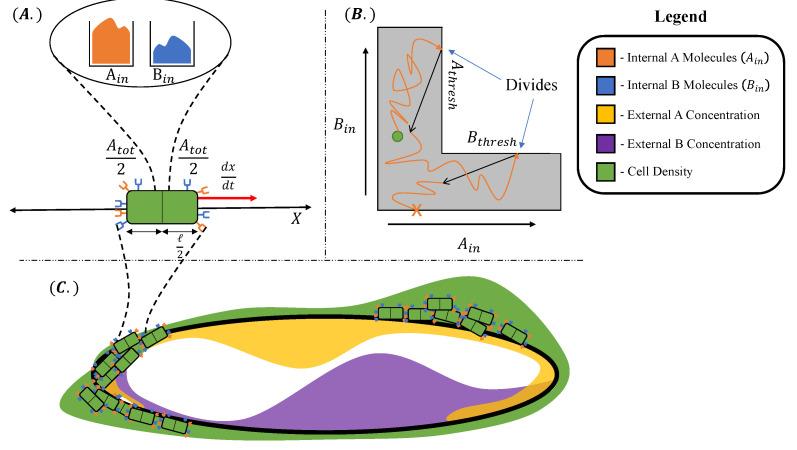
Model components and topology. (**A**) Each individual cell has an internal storage of metabolic resources $A_{\mathrm{in}}$ and $B_{\mathrm{in}}$, and receptors $A_{\mathrm{tot}}/2$ and $B_{\mathrm{tot}}/2$ on the right and $A_{\mathrm{tot}}/2$ and $B_{\mathrm{tot}}/2$ on the left end of the cell. The cell has length *ℓ* and moves through space with velocity dx/dt driven by differences in bound receptors. (**B**) Each cell’s internal resources exist in an *L*-shaped domain given by $\left\{0\leq A_{\mathrm{in}}\leq A_{\mathrm{thresh}},0\leq B_{\mathrm{in}}\right\}\cup \left\{0\leq A_{\mathrm{in}},0\leq B_{\mathrm{in}}\leq B_{\mathrm{thresh}}\right\}$. Starting from an initial resource profile (green dot), the cell accumulates metabolic resources (orange trajectory) until it reaches a cell division boundary $S_{\mathrm{div}}$ (see text for details). Upon reaching $S_{\mathrm{div}}$, the cell divides into two, partitioning its metabolic resources (black arrow). On the contrary, a cell dies if its internal storage of either A or B decreases to zero (orange “X”). (**C**) The geometry of the environment can be thought of as a 1D ring, 0≤x<L. The environmental concentrations of metabolites A(x), B(x) vary with position around the ring.

Our organism is a motile species of cells of length *ℓ*, inhabiting a one-dimensional circular (periodic) environment of length L≫ℓ, for a time t∈[0,T] as illustrated in Figure 1. In particular, our mathematical model is defined according to the following assumptions:

- The cell is rod-like (an abstraction of many motile bacteria), for which we distinguish “right” and “left” ends relative to its position axis.
- The metabolic substrates, denoted A and B, are present in the environment at determinate concentrations for each environment location x∈[0,L). We impose a nonuniform distribution of metabolites A and B, given by local concentration functions A(x) and B(x). For simplicity, we consider these concentrations to be static within the timescale of microbial population growth *T*.
- Each cell at environment location x(t) at time *t* maintains an internal storage of both substrate A and B molecules, i.e., $A_{\mathrm{in}}\left(t\right)$ and $B_{\mathrm{in}}\left(t\right)$, by **absorbing** A and B molecules from the environment proportionally to the concentrations A(x(t)) and B(x(t)), respectively, according to a determinate constant absorption coefficient *k*.
- The cell receives information about its environment by **sensing**, which is realized through the binding of distinct chemical receptor proteins to A and B molecules.
- We endow each cell with a fixed budget of $R_{\mathrm{tot}}$ receptor proteins in total, equally distributed between the right and the left sides. The cell has $A_{\mathrm{tot}}\left(t\right)$ receptors for A molecules and $B_{\mathrm{tot}}\left(t\right)$ receptors for B molecules at time *t*, respectively, where $A_{\mathrm{tot}}\left(t\right)+B_{\mathrm{tot}}\left(t\right)\equiv R_{\mathrm{tot}}$ and $R_{\mathrm{tot}}$ is constant over time.
- The cell reacts to its surroundings by **moving** along the direction of and proportionally to an estimation of the gradients of the concentrations A(x(t)) and B(x(t)) from the numbers of bound receptors for A and B molecules on the right and left sides, denoted as $A_{R}^{*}$, $B_{R}^{*}$, $A_{L}^{*}$, and $B_{L}^{*}$, respectively.
- The cell can control the amount of received information about the concentrations A(x(t)) and B(x(t)) and their gradients by way of **(re-)allocating** the $R_{\mathrm{tot}}$ receptors between $A_{\mathrm{tot}}\left(t\right)$ receptors for A molecules and $B_{\mathrm{tot}}\left(t\right)$ receptors for B molecules. In this paper, we contrast two different strategies, namely, a constant *equal receptor allocation* and an *adaptive receptor allocation*, the latter with the goal of acquiring more information about the more scarce substrate in its internal storage. We make the (strong) assumption that these cells can rapidly convert receptors between A-specific and B-specific types, without incurring a substantive metabolic cost for the transition.
- The cell consumes the substrates from its internal storage with a rate corresponding to a basal maintenance rate of metabolism *S* [26,27]. To grow and divide, a cell must maintain positive internal storage $A_{\mathrm{in}}\left(t\right)$ and $B_{\mathrm{in}}\left(t\right)$. The occurrence of cell division and cell death events are consequently decided by **assessing** the values of the current internal storage $A_{\mathrm{in}}\left(t\right)$ and $B_{\mathrm{in}}\left(t\right)$: when both the amounts of $A_{\mathrm{in}}\left(t\right)$ and $B_{\mathrm{in}}\left(t\right)$ exceed a specified threshold, the cell divides into two daughter cells, each receiving half of the mother cell’s internal storage. If either of the amounts of $A_{\mathrm{in}}\left(t\right)$ and $B_{\mathrm{in}}\left(t\right)$ in a particular cell becomes zero, the cell dies.

According to these assumptions, we express the behavior of a cell according to the following mathematical model.

**Absorbing**. The internal concentrations of A and B, $A_{\mathrm{in}}\left(t\right)$ (respectively $B_{\mathrm{in}}\left(t\right)$), of a cell located at position x(t) evolves according to

(1) $$\frac{dA_{\mathrm{in}}\left(t\right)}{dt}=kA\left(x\left(t\right)\right)-S,$$

where the absorption coefficient *k* [28] translates from concentration to rate of molecules absorbed, and quantifies how easily a cell can absorb molecules. $B_{\mathrm{in}}\left(t\right)$ is computed by substituting B in place of A. The parameter *S* represents a constant metabolic maintenance cost [27], assumed to be the same for both A and B. Table 1 lists the simulation parameters used.

**Sensing**. We model the state of the receptors at time *t* as a sample taken at equilibrium from a binomial binding/release process [22,29]. Thus, the receptor noise is binomial, meaning the numbers of bound receptors satisfy

(2) $$\begin{array}{cc} A_{R}^{*} & ∼\mathrm{Binom}\left(p_{\mathrm{bind},A_{R}},\frac{A_{\mathrm{tot}}}{2}\right),\\ A_{L}^{*} & ∼\mathrm{Binom}\left(p_{\mathrm{bind},A_{L}},\frac{A_{\mathrm{tot}}}{2}\right),\\ B_{R}^{*} & ∼\mathrm{Binom}\left(p_{\mathrm{bind},B_{R}},\frac{B_{\mathrm{tot}}}{2}\right),\\ B_{L}^{*} & ∼\mathrm{Binom}\left(p_{\mathrm{bind},B_{L}},\frac{B_{\mathrm{tot}}}{2}\right), \end{array}\begin{array}{c} p_{\mathrm{bind},A_{R}}=\frac{\left(A\left(x\right)+\frac{\ell }{2}\cdot \nabla A\left(x\right)\right)}{K_{d}+\left(A\left(x\right)+\frac{\ell }{2}\cdot \nabla A\left(x\right)\right)},\\ p_{\mathrm{bind},A_{L}}=\frac{\left(A\left(x\right)-\frac{\ell }{2}\cdot \nabla A\left(x\right)\right)}{K_{d}+\left(A\left(x\right)-\frac{\ell }{2}\cdot \nabla A\left(x\right)\right)},\\ p_{\mathrm{bind},B_{R}}=\frac{\left(B\left(x\right)+\frac{\ell }{2}\cdot \nabla B\left(x\right)\right)}{K_{d}+\left(B\left(x\right)+\frac{\ell }{2}\cdot \nabla B\left(x\right)\right)},\\ p_{\mathrm{bind},B_{L}}=\frac{\left(B\left(x\right)-\frac{\ell }{2}\cdot \nabla B\left(x\right)\right)}{K_{d}+\left(B\left(x\right)-\frac{\ell }{2}\cdot \nabla B\left(x\right)\right)}, \end{array}$$

where $p_{\mathrm{bind},A_{R}}$ is the probability of binding A molecules for a single receptor at the right side of the cell, $A\left(x\right)\pm \frac{\ell }{2}\cdot \nabla A\left(x\right)$ is the the local concentration of A (resp. B) at the right and left end of the cell, *∇* is the gradient operator (here, $\frac{d}{dx}$), and $K_{d}$ is the chemical dissociation constant of the receptor [30]. For simplicity, we omitted the time variable *t*, we presume that samples taken at successive time steps and at opposite sides of the cell are independent, and we assume the same dissociation constant for each receptor.

**Moving**. We set the cell velocity to be proportional to the difference between the number of receptors bound on the right versus left sides of the cell:

(3) $$\begin{array}{c} \frac{dx}{dt}=v\left(\frac{2v_{\max}}{1+e^{-\left(A_{R}^{*}+B_{R}^{*}-A_{L}^{*}-B_{L}^{*}\right)}}-v_{\max}\right)\;, \end{array}$$

where *v* is a constant parameter that converts a number of bound receptors to velocity, and $v_{\max}$ corresponds to the maximum velocity.

**(Re-)allocating**. The *equal receptor allocation* strategy seeks the greatest possible information about the environmental concentrations A(x) and B(x), regardless of their importance for cell survival, by setting a static distribution $A_{\mathrm{tot}}=B_{\mathrm{tot}}$ for all *t*, regardless of the cell’s internal storage of A and B molecules.

The *adaptive receptor allocation* strategy, in contrast, attempts to increase the probability of survival for the cell by distributing receptors among the two types in a way that takes into account $B_{\mathrm{in}}\left(t\right)$ and $A_{\mathrm{in}}\left(t\right)$. The receptor apportionment rule is given as follows:

$$\begin{array}{c} B_{\mathrm{tot}}\left(t\right)=R_{\mathrm{tot}}\left(\frac{A_{\mathrm{in}}\left(t\right)}{A_{\mathrm{in}}\left(t\right)+B_{\mathrm{in}}\left(t\right)}\right)\;, \end{array}$$

and $A_{\mathrm{tot}}\left(t\right)=R_{\mathrm{tot}}-B_{\mathrm{tot}}\left(t\right)$. In this way, the cell redistributes its receptors in proportion to its relative deficit of one metabolite versus the other. For example, if the cell has fewer B than A molecules, it will move a greater portion of its receptors to receptor type B. It is this apportionment rule that implements the “subjectivity” of information reception in this model, which is reflected in the simulation results in Section 5.

**Assessing**. A cell divides if its internal metabolites $A_{\mathrm{in}}\left(t\right)$ and $B_{\mathrm{in}}\left(t\right)$ both surpass a division threshold $A_{\mathrm{thresh}}=B_{\mathrm{thresh}}=D$. We define the cell division time $\tau_{\mathrm{div}}$ to be the first passage time of the cell’s $\left(A_{\mathrm{in}}\left(t\right),B_{\mathrm{in}}\left(t\right)\right)$ trajectory to the cell division “surface” $S_{\mathrm{div}}\equiv S_{A}\cup S_{B}$, where

(4) $$\begin{array}{cc} S_{A} & =\{\left(a,b\right)\;|\;a=A_{\mathrm{thresh}},\;b\geq B_{\mathrm{thresh}}\}\\ S_{B} & =\{\left(a,b\right)\;|\;a\geq A_{\mathrm{thresh}},\;b=B_{\mathrm{thresh}}\}. \end{array}$$

Figure 1B illustrates the division surface. Thus, as $t\to \tau_{\mathrm{div}}$, the cell’s internal state approaches $\left(A_{\mathrm{in}}^{*},B_{\mathrm{in}}^{*}\right)\in S_{\mathrm{div}}$. At time $\tau_{\mathrm{div}}$, the old cell is replaced with two daughter cells at the same current location, each with internal state

(5) $$\left(A_{\mathrm{in}}\left(\tau_{\mathrm{div}}^{+}\right),B_{\mathrm{in}}\left(\tau_{\mathrm{div}}^{+}\right)\right)=\left(\frac{A_{\mathrm{in}}^{*}}{2},\frac{B_{\mathrm{in}}^{*}}{2}\right),$$

and increment the total population count by one. The two daughter cells then move independently of one another.

A cell dies if its internal storage $\left(A_{\mathrm{in}}\left(t\right),B_{\mathrm{in}}\left(t\right)\right)$ reaches the death threshold $S_{\mathrm{death}}\equiv \left\{\left(a,b\right)\;|\;a=0\;\mathrm{or}\;b=0\right\}$. In this case, the old cell ceases to exist, and the total population count is decremented by one.

### 2.2. Computational Model

To appreciate the emergence of “subjective information” and its correlation with organism survival in the mathematical abstraction detailed in Section 2.1, we derive here a computational model that is later utilized to obtain numerical simulation results.

In the rest of this section, we consider a sampled environment [0,L] so that each discrete location in the environment $\bar{x}\in \left\{i\;L/N\;|\;i=0,\ldots ,N-1\right\}$. Here, we impose periodic boundary conditions, where $\bar{x}=0$ (i=0) and $\bar{x}=L$ (i=N) represent the same location. Likewise, we sample the time variable into $\bar{t}\in \left\{j\;\Delta t\;|\;j=0,\ldots ,T/\left(\Delta t\right)\right\}$.

**Figure 2:**
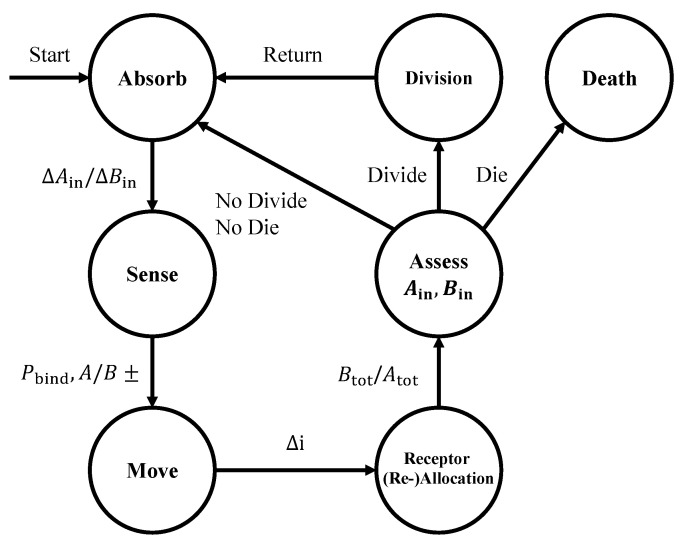
State machine diagram of the cell computational model formulated in this paper. See text for details.

In Figure 2, we show a state machine diagram of our computational model, which is based on the definition and the cell behavior abstractions presented in Section 2.1. In particular, the discrete states of the cell and the transitions that may occur within a time sample Δt at time $\bar{t}$ include the following: **absorption** of A and B molecules, resulting into the changes $\Delta A_{\mathrm{in}}\left(\bar{t}\right)$ and $\Delta B_{\mathrm{in}}\left(\bar{t}\right)$ in the internal storage of A and B molecules, respectively; **sensing** through the chemical receptors, which results into the numbers of bound receptors for A and B molecules, i.e., $A_{R}^{*}$, $B_{R}^{*}$, $A_{L}^{*}$, and $B_{L}^{*}$, respectively, according to (2); **movement** by a discrete length Δi based on the estimated concentration gradients, and received information modulation via **receptor (re-)allocation**, which follows one of the two aforementioned strategies to allocate $A_{\mathrm{tot}}\left(\bar{t}+\Delta t\right)$ and $B_{\mathrm{tot}}\left(\bar{t}+\Delta t\right)$. Finally, the cell divides if it **assesses** a surplus of molecule A and B that both exceed the threshold *D*, or dies if its internal storage $\left(A_{\mathrm{in}}\left(\bar{t}\right),B_{\mathrm{in}}\left(\bar{t}\right)\right)$ reaches the death threshold $S_{\mathrm{death}}$. Each of the aforementioned states reproduces the cell behaviors detailed in Section 2.1 as follows.

**Absorb.** The cell absorbs A and B molecules according to the average of the external concentrations A(x) and B(x) at the left and right of the cell, $\bar{x}\left(\bar{t}\right)-\frac{\ell }{2}$ and $\bar{x}\left(\bar{t}\right)+\frac{\ell }{2}$, respectively. To compute the absorbed A (or B) molecules $\Delta A_{\mathrm{in}}\left(\bar{t}\right)$ (or $\Delta B_{\mathrm{in}}\left(\bar{t}\right)$), Equation (1) is changed into

(6) $$\Delta A_{\mathrm{in}}\left(\bar{t}\right)=\left\lfloor \left(k\frac{A\left(\bar{x}\left(\bar{t}\right)-\frac{\ell }{2}\right)+A\left(\bar{x}\left(\bar{t}\right)+\frac{\ell }{2}\right)}{2}-S\right)\Delta t\right\rfloor \;,$$

where $\Delta B_{\mathrm{in}}$ is computed by substituting B in place of A. $\Delta A_{\mathrm{in}}$ and $\Delta B_{\mathrm{in}}$ are then added to the internal storage of A and B molecules to obtain $A_{\mathrm{in}}\left(\bar{t}\right)$ and $B_{\mathrm{in}}\left(\bar{t}\right)$, respectively. If the resulting $A_{\mathrm{in}}\left(\bar{t}\right)$ and $B_{\mathrm{in}}\left(\bar{t}\right)$ are negative, then they are set to zero independently and cell death is assessed later on.

**Sense.** The state of the receptors at time $\bar{t}$ is a result of the same Binomial random processes as in (2), where this time we define the probability of binding A molecules at the right side of the cell as follows:

(7) $$p_{\mathrm{bind},A_{R}}=\frac{A(\bar{x}+\frac{\ell }{2})}{K_{d}+A\left(\bar{x}+\frac{\ell }{2}\right)}\;,$$

Similarly, we define the probability $p_{\mathrm{bind},A_{L}}$ of binding A molecules for a single receptor at the left end of the cell (at location $\bar{x}-\frac{\ell }{2}$), with analogous definitions for the probabilities $p_{\mathrm{bind},B_{R}}$ and $p_{\mathrm{bind},B_{L}}$ for the B receptors. We assume in (7) that the time step Δt of the simulation is long enough that the receptors and surrounding concentration reach a steady state.

**Move.** We express the cell movement at time $\bar{t}$ according to an estimate of the local gradient of the concentration of molecules of type A and B, similarly as in (3):

(8) $$\begin{array}{c} \psi =A_{R}^{*}+B_{R}^{*}-A_{L}^{*}-B_{L}^{*}\\ \Delta i=\left\{\begin{array}{cc} -v_{\max} & \psi \leq -v_{\max}\\ \psi & -v_{\max}\leq \psi \leq v_{\max}\\ v_{\max} & \psi \geq v_{\max} \end{array}\right. \end{array}$$

where Δi is the change in cell location, corresponding to $\Delta \bar{x}=\Delta i\;L/N$, and $v_{\max}$ is the maximum allowed movement in a single step.

**Receptor (Re-)Allocation**. In the *equal receptor allocation* strategy, the cell behaves as detailed in Section 2.1. In the *adaptive receptor allocation* strategy, at time $\bar{t}$, the cell redistributes the receptors that will be considered in the absorption and sensing at time $\bar{t}+\Delta t$ among the two types based on the ratio of A to B molecules internal to the cell as follows:

(9) $$B_{\mathrm{tot}}\left(\bar{t}+\Delta t\right)=2\left\lfloor \frac{R_{\mathrm{tot}}}{2}\frac{A_{\mathrm{in}}\left(\bar{t}\right)}{A_{\mathrm{in}}\left(\bar{t}\right)+B_{\mathrm{in}}\left(\bar{t}\right)}\right\rfloor \;,$$

where $B_{\mathrm{tot}}\left(\bar{t}+\Delta t\right)$ is the new B-receptor count, $A_{\mathrm{in}}\left(\bar{t}\right)$, $B_{\mathrm{in}}\left(\bar{t}\right)$ are the internal A, B molecule storage after the cell absorption, and $A_{\mathrm{tot}}\left(\bar{t}+\Delta t\right)=R_{\mathrm{tot}}-B_{\mathrm{tot}}\left(\bar{t}+\Delta t\right)$

**Assess**. The cell moves to the Divide state if the internal molecule numbers $A_{\mathrm{in}}\left(\bar{t}\right)$ and $B_{\mathrm{in}}\left(\bar{t}\right)$ both exceed the division threshold *D* or, equivalently, if the trajectory of $\left(A_{\mathrm{in}}\left(\bar{t}\right),B_{\mathrm{in}}\left(\bar{t}\right)\right)$ has crossed the division surface $S_{\mathrm{div}}$, as defined in Section 2.1 and (4). The threshold *D* is set to the minimum energetic requirement needed in both A and B molecule storage for the cell to survive five time steps. If either of the internal molecule numbers $A_{\mathrm{in}}\left(\bar{t}\right)$ and $B_{\mathrm{in}}\left(\bar{t}\right)$ is equal to zero, the cell moves to the Death state. If a cell does not divide or die, then its state moves back to Absorb, and the process is repeated.

**Divide.** In this state, the cell loses half of $A_{\mathrm{in}}$ and $B_{\mathrm{in}}$ and an identical daughter cell is created at the same position $\bar{x}$ with an equal amount of $A_{\mathrm{in}}\left(\bar{t}+\Delta t\right)$ and $B_{\mathrm{in}}\left(\bar{t}+\Delta t\right)$ in the subsequent time step. The cell subsequently transitions to the Absorb state.

**Death.** In this state, the cell is considered dead and will be removed from the environment.

**Figure 3:**
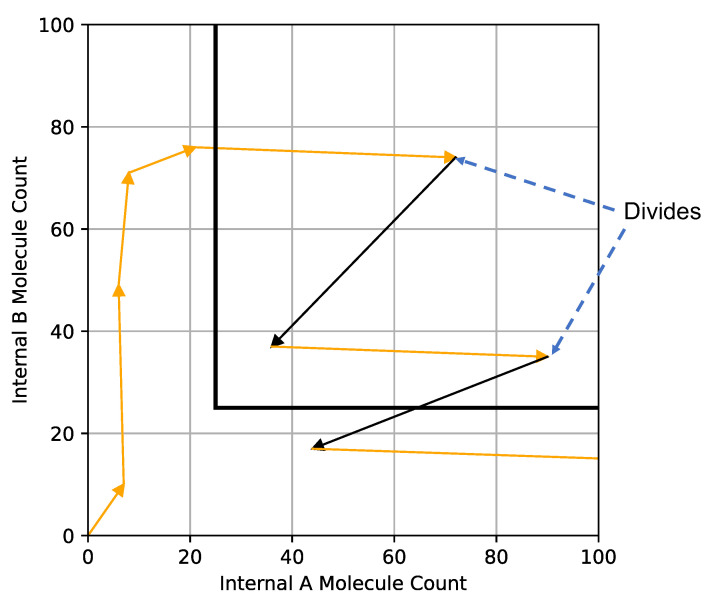
Trajectory of a simulated cell projected onto the $A_{\mathrm{in}}$ and $B_{\mathrm{in}}$ plane. The black line defines the threshold at which the cell can divide, and upon which it loses half its $A_{\mathrm{in}}$ and $B_{\mathrm{in}}$ to its daughter cell. The black arrows define cell division and the cell’s internal state when division takes place. In this example, after one cell division (upper black arrow), the cell has enough $A_{\mathrm{in}}$ and $B_{\mathrm{in}}$ to divide again on the next timestep (lower black arrow).

Figure 3 shows an example of the trajectory of $\left(A_{\mathrm{in}}\left(\bar{t}\right),B_{\mathrm{in}}\left(\bar{t}\right)\right)$ in a simulation of the computational model. In this example, each time step is represented by an orange arrow and cell divisions by black arrows. The cell starts to gather $B_{\mathrm{in}}$, moves through the divide threshold, and proceeds to divide twice in two time steps and ends with a higher $A_{\mathrm{in}}$ count below the division threshold. (This example shows how in a discrete computational model the cell could exist beyond the division threshold for multiple time steps.)

The numerical results presented in Section 5 are obtained by simulating this computational model according to the parameter values listed in Table 1.

## 3. Performance Metrics

We compare the two receptor allocation strategies using two metrics: one based on the amount of information cells acquire from the environment, and the other based on the cell growth. The former is formulated as the average mutual information [5] between the input environmental concentration of A and B molecules and the output number of bound receptors $A_{R}^{*}$, $B_{R}^{*}$, $A_{L}^{*}$, and $B_{R}^{*}$, which are then used by the cell to estimate their gradients. The latter is expressed as the cell growth rate, which quantifies the exponential growth rate of the cells by utilizing the resources in the environment.

**Information Efficiency**. The Mutual Information (MI) formula is expressed as follows [5]:

(10) $$MI_{X,Y}=\sum_{y\in Y}\sum_{x\in X}P_{\left(X,Y\right)}\left(x,y\right)\log_{2}\frac{P_{\left(X,Y\right)}\left(x,y\right)}{P_{X}\left(x\right)P_{Y}\left(y\right)},$$

where *X* is the set of input values, *Y* is the set of output values, $P_{X}\left(x\right)$ and $P_{Y}\left(y\right)$ are the marginal probabilities of *X* and *Y*, and $P_{\left(X,Y\right)}\left(x,y\right)$ is the joint probability of *X* and *Y*, respectively. The exact MI calculation in the simulation given a set of input and output data is defined in Algorithms 1–3.
**Algorithm 1:** Entropy (*X*)
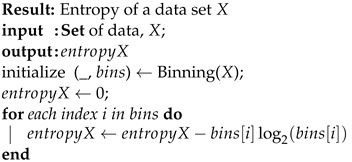

**Algorithm 2:**MI(*X*,*Y*)
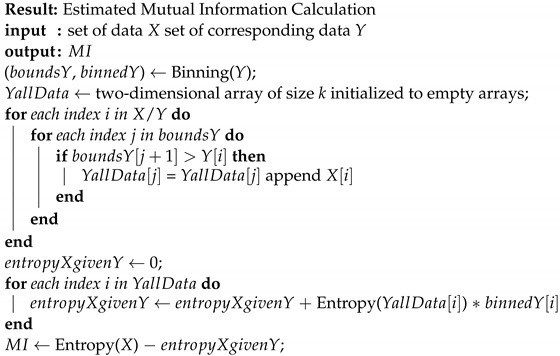

**Algorithm 3:** Binning (*Y*)
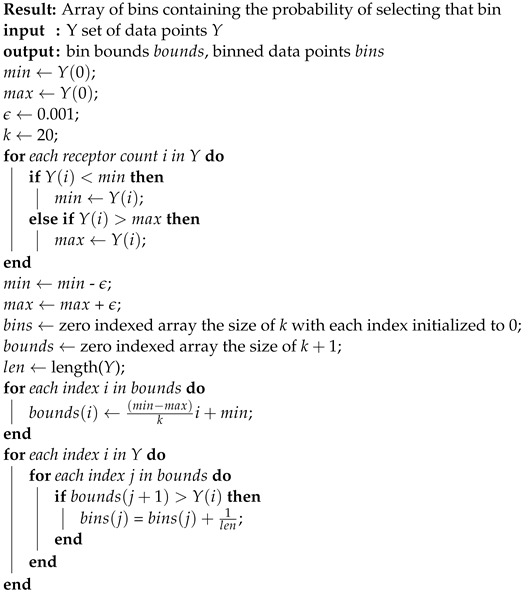


The average MI at time *t* (MI(t)) of the cells in the environment is defined as

(11) $$MI\left(t\right)=E_{\mathrm{Total}\#\mathrm{of}\mathrm{cells},t}\left[MI_{cell}\right]\;,$$

where $E_{\mathrm{Total}\#\mathrm{of}\mathrm{cells},t}\left[\cdot \right]$ denotes the average computed over the entire population of cells present in the environment and over time up to time *t*, and $MI_{cell}$ is the MI of a cell, computed as follows:

(12) $$\begin{array}{cc} MI_{cell} & =MI_{A_{R},A_{R}^{*}}+MI_{B_{R},B_{R}^{*}}+MI_{A_{L},A_{L}^{*}}+MI_{B_{L},B_{L}^{*}}\;, \end{array}$$

where $A_{R}=A\left(\bar{x}+\frac{\ell }{2}\right)$, $B_{R}=B\left(\bar{x}+\frac{\ell }{2}\right)$, $A_{L}=A\left(\bar{x}-\frac{\ell }{2}\right)$, and $B_{L}=B\left(\bar{x}-\frac{\ell }{2}\right)$ are the environmental concentrations of molecules A or B on the right and left of a cell, respectively. The MI of the individual channels between the external concentration and bound receptors are assumed to be uncorrelated and are therefore added together. To express (10), we assume that each of the environmental concentrations and consequent number of bound receptors are independent from each other [30].

**Growth Rate**. We introduce death into the model and simulation to make the growth of the cells more realistic representations of in vivo environments. The growth rate over a time *T* is defined here in the same way as growth is defined in other systems, namely as the exponential rate of growth, that is, the doubling rate (or multiplication rate) over time,

(13) $$\begin{array}{c} G_{T}=\frac{\Delta t}{T}\sum_{j=0}^{(T/\Delta t)-1}\log_{2}\left(\frac{P_{j+1}}{P_{j}}\right), \end{array}$$

where $P_{j}$ is the population size at the end of the discrete time step with index *j*, as defined in Section 2.2. To obtain the growth rate results presented in Section 5, we discarded an initial transient and evaluated $P_{j+1}/P_{j}$ by sampling from an ensemble at equilibrium.

## 4. Simulation Implementation

We simulate the model from Section 2.2 with many cells and their interaction with the environment using a computer program. The simulation results presented are obtained for different initial conditions and simulation parameters. The simulation is divided into time steps, where each step *j* is a cycle of the state machine. The main simulation parameters with their description and initialization values are shown in Table 1. Additional choices made for this simulation are as follows.

**Table 1:** Simulation parameters.

Variable	Description	Initialization
$A_{\mathrm{tot}},B_{\mathrm{tot}}$	Total A/B receptor count	200 receptors
$A\left(\bar{x}\right),B\left(\bar{x}\right)$	A/B external concentrations at location $\bar{x}$	0
$A_{R}^{*},A_{L}^{*},B_{R}^{*},B_{L}^{*}$	A/B left/right bound receptor count	0
$R_{\mathrm{tot}}$	Total number of receptors for a cell to allocate	400 receptors
$A_{\mathrm{in}},B_{\mathrm{in}}$	Internal A and B molecule count	0
*i*	Cell Location	[51, 1–100]
*k*	Absorption Coefficient	[1.0–5.0]
$K_{d}$	Dissociation Constant	2.0
*S*	Basal Energetic Requirement	[1,5]
*D*	Division Threshold	5S
T/Δt	Total Simulation Time Steps	[30–100]
$v_{\max}$	Max Cell Velocity	10
$Cell_{\max}$	Max Cell Count	[2000, 10,000]
$Cell_{\mathrm{adjusted}}$	Adjusted Cell Count	[1000,9000]
$Cell_{\mathrm{multi}}$	Cell Multiplier	1

The cells’ environment consists of discrete locations that define the external concentration and the position of the cells. The cells have length ℓ=2, with the left and right defined as being the current position minus one and the current position plus one, respectively. The simulation is optimized for limited computational resources through approximation of the real cell count in the environment by keeping the max number of environment cells lower than $Cell_{\max}$ during any single time step. If $Cell_{\max}$ is surpassed in a given time step, the number of cells at each location is reduced, and individual cells are removed at random so that the total number of environment cells is equal to a lower cell count $Cell_{\mathrm{adjusted}}$, as defined by the system parameters in Table 1. These decrements of the total population are carried out in such a way that the overall spatial distribution of cells remains the same. A running multiplier $Cell_{\mathrm{multi}}$ is then iterated upon, by multiplying it with the ratio between the initial high cell count and $Cell_{\mathrm{adjusted}}$. A real cell count is then calculated by multiplying the current number of environmental cells by this multiplier. The cell stress $c_{*}=S/k$ is defined here as the of the metabolic cost *S* to the substrate absorption coefficient *k*, as defined in Section 2.1. Heuristically speaking, the cell stress parameter $c_{*}$ represents the average concentration of either substrate that a cell would have to experience along its spatial trajectory, in order to maintain a net gain in metabolites (cf. (1)).

We employ a non-uniform distribution of A and B molecule concentrations in order for the cells to sense and respond to spatial differences in substrate concentrations. The concentrations of molecular substrates A and B, which remain static throughout the simulation time despite cell intake, are given by the von Mises distribution as

(14) $${\left[C\right]}_{i}={\left[C\right]}_{\max}\exp\left[\kappa_{C}\frac{\cos(2\pi \left({\bar{x}}_{i}-\mu_{C}\right)/L)}{\left(L\;I_{0}\left(\kappa_{C}\right)\right)}\right],$$

where C∈{A,B}, $\mu_{A}=25$ and $\mu_{B}=75$, respectively, $\kappa_{A}=\kappa_{B}=0.1$, and $I_{0}$ is the modified Bessel function of the first kind [31]. We choose the von Mises distribution because it is the maximal entropy distribution on a periodic support for a given mean and circular variance [32]. ${\left[A\right]}_{\max}={\left[B\right]}_{\max}=500$ for all simulations and they remain static over time and do not change in response to the cell’s absorption process. The nonuniform distribution of molecules of type A and B across the environment results in a non-zero expected gradient in the number of bound receptors along a cell. Therefore, cells can use chemotaxis to improve their food intake, setting the stage for a non-trivial analysis of the two receptor allocation strategies.

## 5. Numerical Results

In the following, we present the numerical results obtained by running the simulations described in Section 4, based on the computational model detailed in Section 2.2, and computing the metrics defined in Section 3 from the obtained data.

**Figure 4:**
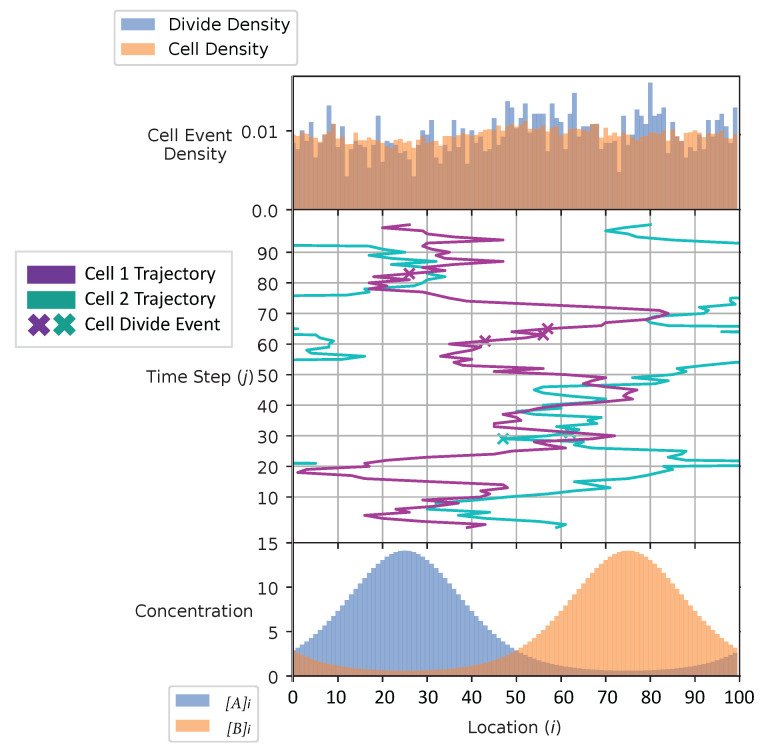
Simulation environment of the equal receptor allocation strategy.

**Figure 5:**
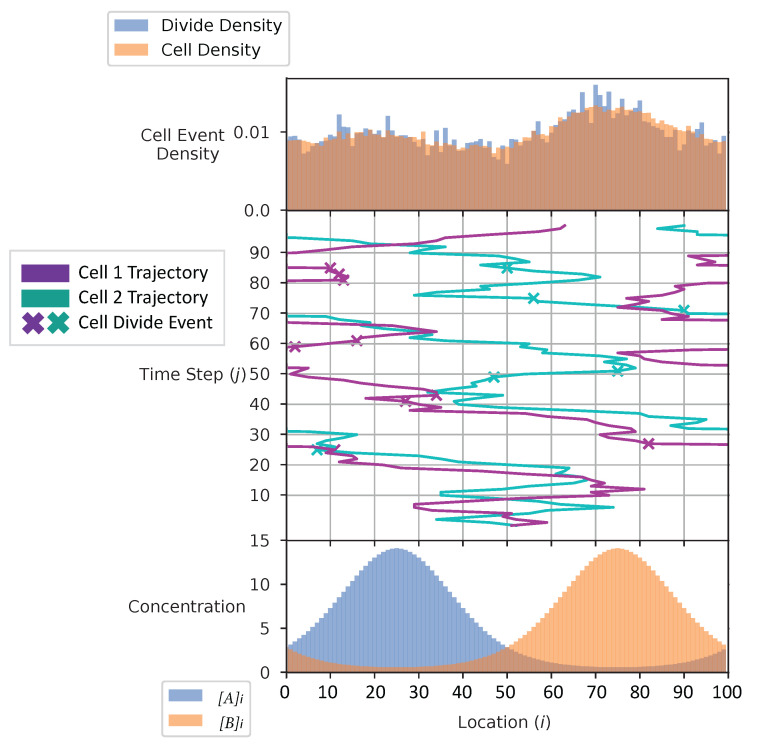
Simulation environment of the adaptive receptor allocation strategy.

In Figure 4 and Figure 5, we show the cell’s environment for the equal and the adaptive receptor allocation strategies, respectively. The lower plot of either figure shows the A and B external concentrations following the von Mises distribution for the entire simulation space *L*. The center of the figures show two cells, each represented by a different color, moving left or right in the environment space. ‘x’ is used here to represent if a cell is divided in a given time step. The simulation for this figure is based on the parameters from Table 1, where *i* is equal to [1–100], *k* is equal to 3.0, *S* is equal to 5, $Cell_{\max}$ is equal to 2000, $Cell_{\mathrm{adjusted}}$ is equal to 1000, and T/Δt is equal to 100 time steps. The upper plot of the figures represents the division density, where the cells divide, and the cell density, where the cells are. The cell’s distribution statistics were compiled from 100 time steps and an initial cell at each location. Here, the cells have an infinite $v_{\max}$.

**Figure 6:**
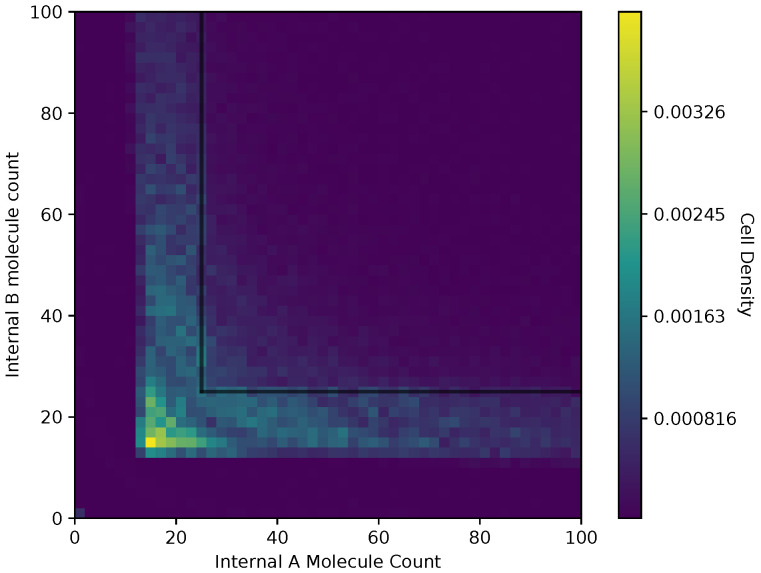
Cell density in $A_{\mathrm{in}}$, $B_{\mathrm{in}}$ space of the equal receptor allocation strategy.

**Figure 7:**
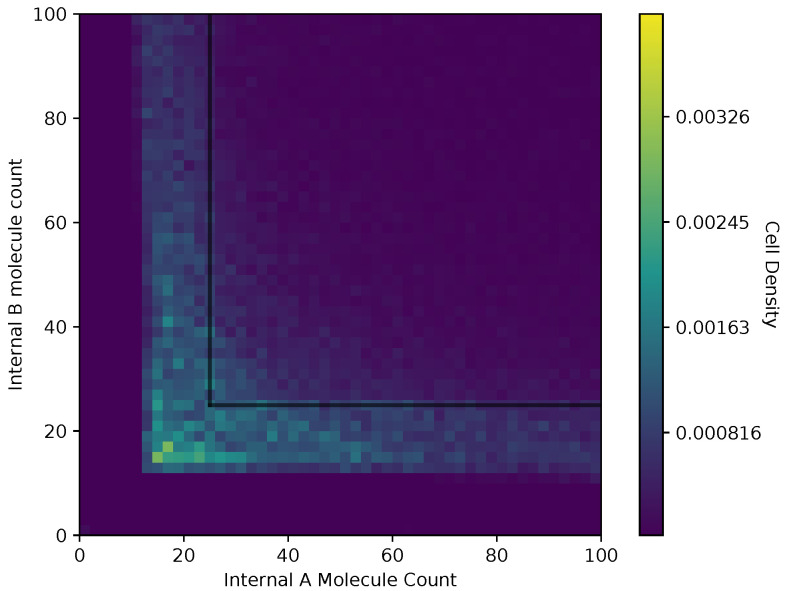
Cell density in $A_{\mathrm{in}}$, $B_{\mathrm{in}}$ space of the adaptive receptor allocation strategy.

In Figure 6 and Figure 7, we show a heat map of the cell density in the $A_{\mathrm{in}}$ and $B_{\mathrm{in}}$ plane for equal and adaptive receptor allocation strategies, respectively. A black line that runs from 25 to 200 in each direction represents the cell division surface $S_{\mathrm{div}}$, as defined in Section 2.1. The simulation parameters used in these figures are the same as in Table 1, specifically with *i* equal to [1–100], *k* equal to 5, *S* equal to 5, $Cell_{\max}$ equal to 10,000, $Cell_{\mathrm{adjusted}}$ equal to 9000, and T/Δt equal to 30. Here, the figure is split into a two-dimensional bin matrix where the corresponding color of each bin represents the portion of cells corresponding to the bin. The color representations are consistent between Figure 6 and Figure 7 to the same portion of cells in each figure. Internal resource levels $A_{\mathrm{in}}$ and $B_{\mathrm{in}}$ are recorded in each time step once before the cell has a chance to divide, but after the absorption state, and again after the cell has a chance to divide. This method allows for a better visualization of the internal state of the cell.

**Figure 8:**
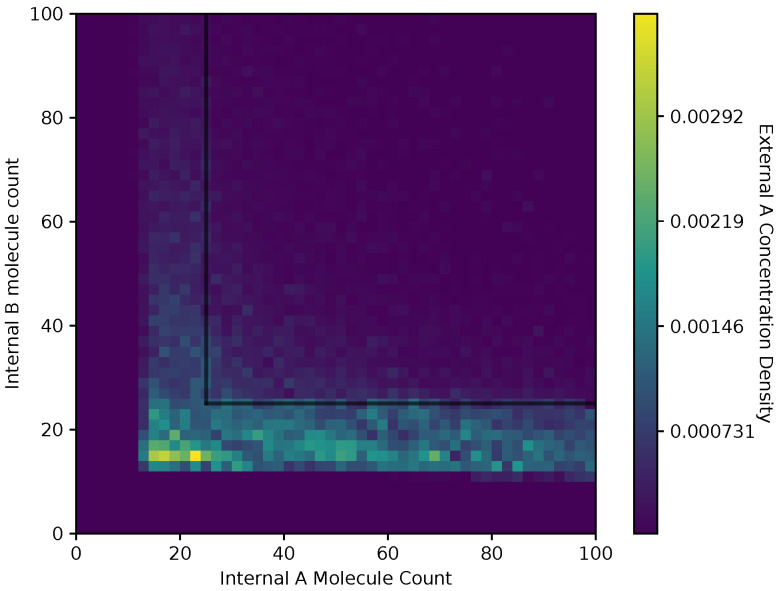
Heat map of the external A concentration with respect to $A_{\mathrm{in}}$ $B_{\mathrm{in}}$ in the equal receptor allocation strategy.

**Figure 9:**
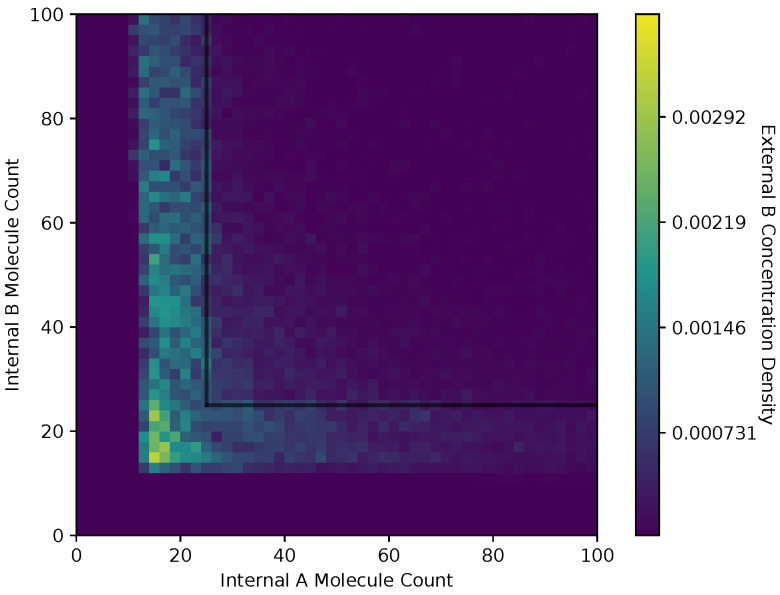
Heat map of the external B concentration with respect to $A_{\mathrm{in}}$ $B_{\mathrm{in}}$ in the equal receptor allocation strategy.

In Figure 8 and Figure 9, we show a heat map of the $A(\bar{x})$ and $B(\bar{x})$, respectively, given $A_{\mathrm{in}}$ and $B_{\mathrm{in}}$, for the equal receptor allocation strategy. The simulation parameters used here are the same as Figure 6 and Figure 7. Here, each bin in the figures represents the portion of $A(\bar{x})$ or $B(\bar{x})$ weighted on how many visits a cell makes to location $\bar{x}$. This figure gives some insight into where the average cell is when it has some internal state. As in Figure 6 and Figure 7, $A(\bar{x})$ and $B(\bar{x})$ are recorded once before the cell can divide, and again after the cell has a chance to divide.

**Figure 10:**
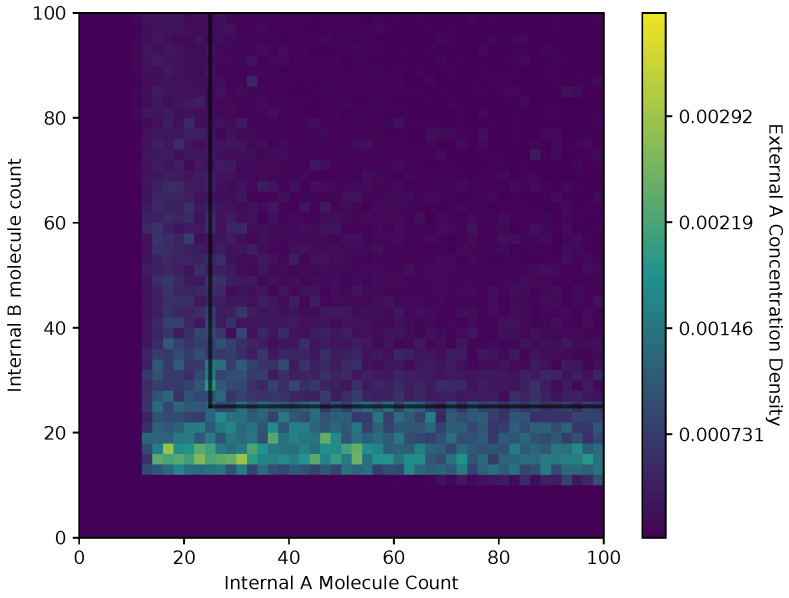
Heat map of the external A concentration with respect to $A_{\mathrm{in}}$ $B_{\mathrm{in}}$ for the adaptive receptor allocation strategy.

**Figure 11:**
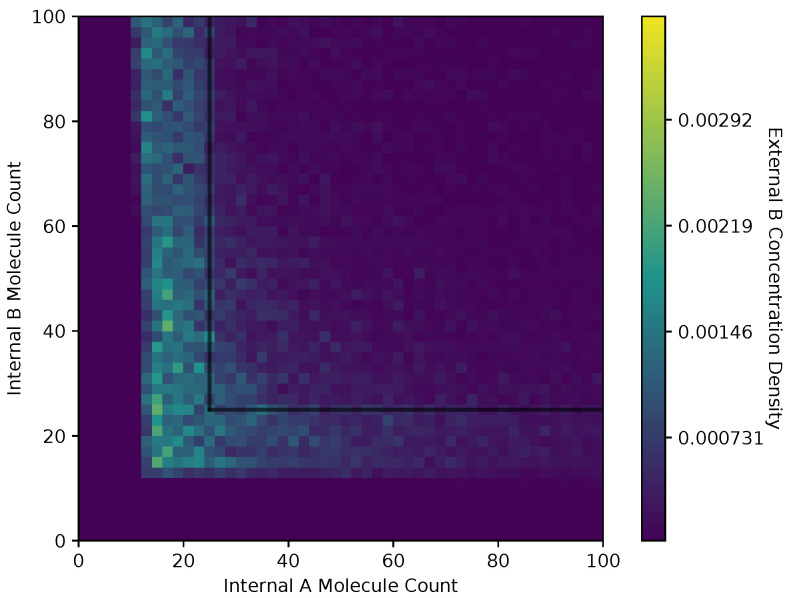
Heat map of the external B concentration with respect to $A_{\mathrm{in}}$ $B_{\mathrm{in}}$ for the adaptive receptor allocation strategy.

In Figure 10 and Figure 11, we show a heat map of the $A(\bar{x})$ and $B(\bar{x})$, respectively, given $A_{\mathrm{in}}$ and $B_{\mathrm{in}}$, for the adaptive receptor allocation strategy. These figures use the same simulation parameters as Figure 6 and Figure 7. The color coding of the densities of $A(\bar{x})$ and $B(\bar{x})$ is consistent across Figure 8, Figure 9, Figure 10 and Figure 11.

**Figure 12:**
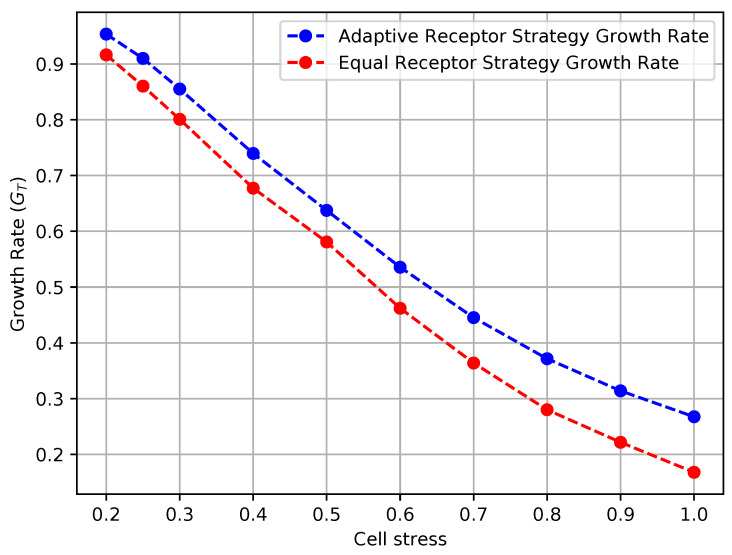
Growth rate of the receptor strategies with respect to cell stress.

**Figure 13:**
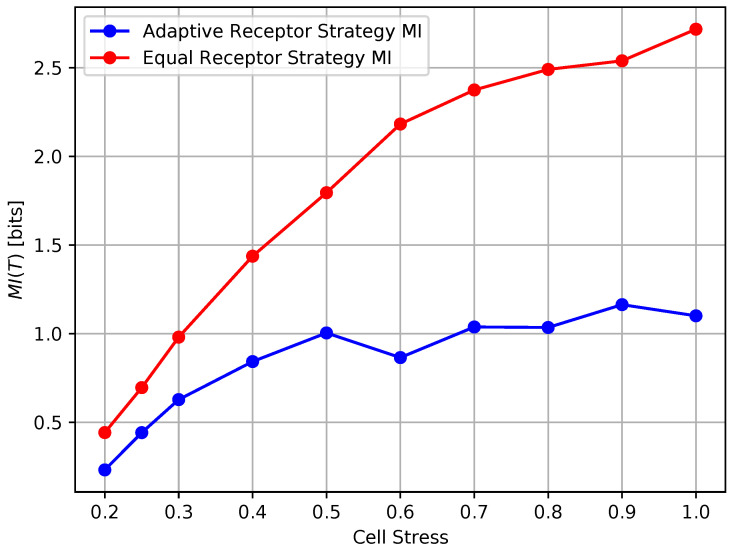
Mutual information of the receptor strategies with respect to cell stress.

In Figure 12 and Figure 13, we show the growth rate $G_{T}$ of the cells and the average MI MI(T), respectively, as defined in Section 3, for the equal receptor allocation strategy and the adaptive receptor allocation strategy as a function of the cell stress $c_{*}$. The simulation parameters used to obtain these results are the same as in Table 1, where *i* is equal to [1–100], *S* is equal to 1, for $Cell_{\max}$ equal to 10,000, $Cell_{\mathrm{adjusted}}$ equal to 9000, and T/Δt equal to 31. The growth rate $G_{T}$ was found in another simulation with the same parameters, where the cell’s movement was sampled from a uniform random distribution between $-v_{\max}$ and $v_{\max}$ at cell stress $c_{*}=1$ This growth rate was found to be 0.17 slightly below the equal receptor strategy.

**Figure 14:**
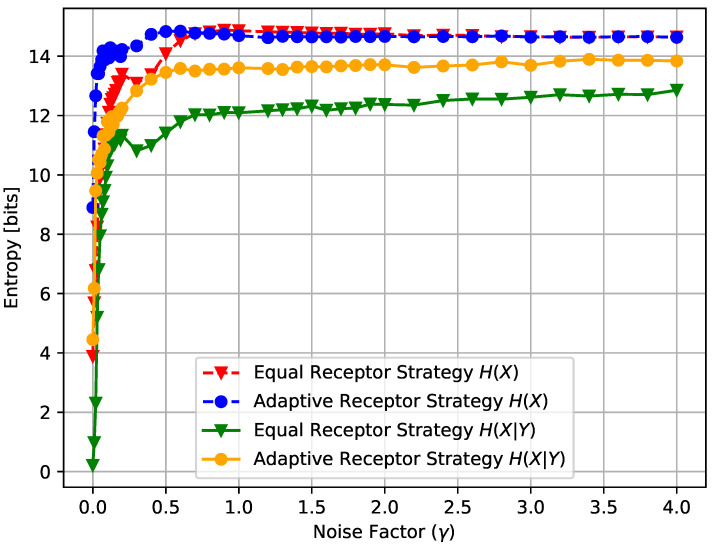
H(X) and H(X|Y) for the equal receptor and adaptive receptor strategy.

**Figure 15:**
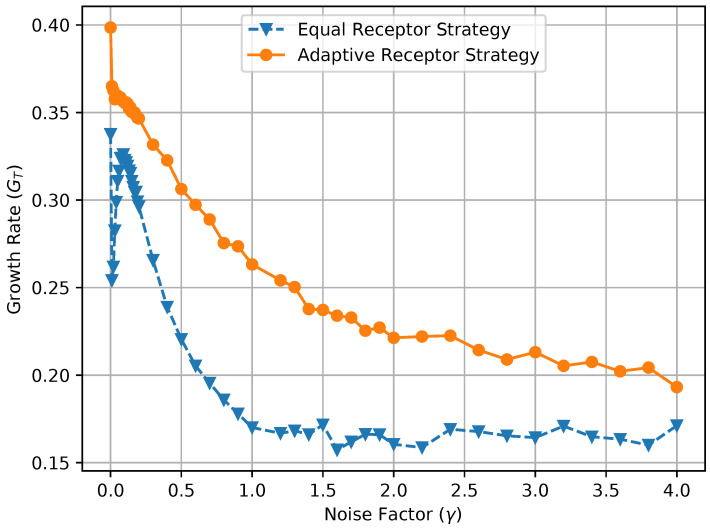
Growth of the equal receptor strategy and adaptive receptor strategy with respect to receptor noise.

**Figure 16:**
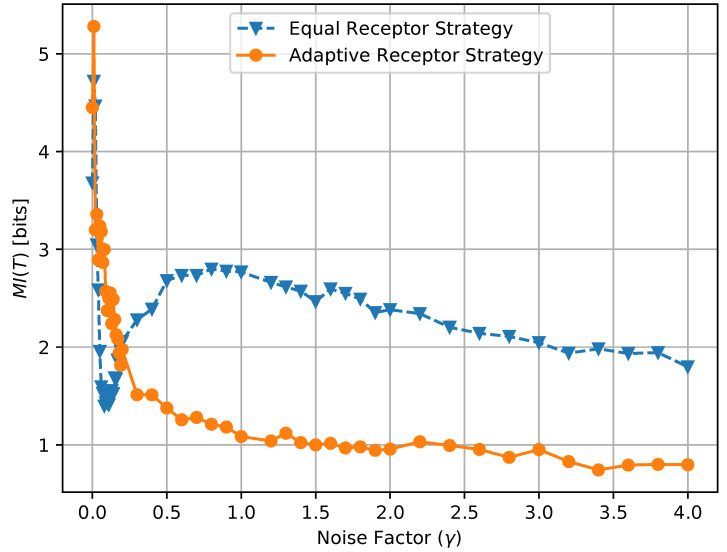
MI(T) of the equal receptor strategy and the adaptive receptor strategy with respect to receptor noise.

**Figure 17:**
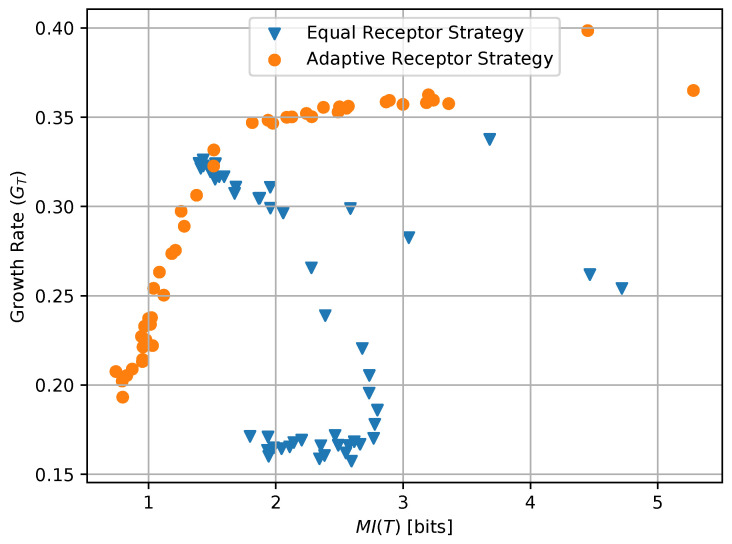
Growth of the equal receptor strategy and the adaptive receptor strategy with respect to the MI(T) as the receptor noise is increased.

In Figure 14, Figure 15 and Figure 16, we show the average input entropy $E_{\#\mathrm{of}\mathrm{cells},T}\left[H_{cell}\left(X\right)\right]$ and average conditional entropy $E_{\#\mathrm{of}\mathrm{cells},T}\left[H_{cell}\left(X|Y\right)\right]$, the growth rate $G_{T}$ of the cells, and the average MI MI(T), respectively, of the Equal Receptor and Adaptive Receptor strategies as a function of the noise factor γ. The input entropy $H_{cell}\left(X\right)$ and conditional entropy $H_{cell}\left(X|Y\right)$ of a cell are defined as follows:

(15) $$\begin{array}{ccc} H(X) & = & -\sum_{x\in X}P_{X}\left(x\right)log_{2}P_{X}\left(x\right)\;, \end{array}$$

(16) $$\begin{array}{ccc} H(X|Y) & = & -\sum_{x\in X}\sum_{y\in Y}P_{X,Y}\left(x,y\right)log_{2}\frac{P_{X,Y}\left(x,y\right)}{P_{Y}\left(y\right)}\;, \end{array}$$

(17) $$\begin{array}{ccc} H_{cell}\left(X\right) & = & H\left(A_{R}\right)+H\left(B_{R}\right)+H\left(A_{L}\right)+H\left(B_{L}\right)\;, \end{array}$$

(18) $$\begin{array}{ccc} H_{cell}\left(X|Y\right) & = & H\left(A_{R}|A_{R}^{*}\right)+H\left(B_{R}|B_{R}^{*}\right)+H\left(A_{L}|A_{L}^{*}\right)+H\left(B_{L}|B_{L}^{*}\right)\;, \end{array}$$

where $A_{R}$, $B_{R}$, $A_{L}$, and $B_{L}$ are the environmental concentrations of molecules A or B on the right and left of a cell, respectively, and $A_{R}^{*}$, $B_{R}^{*}$, $A_{L}^{*}$, and $B_{R}^{*}$ are the consequent output number of bound receptors on the cell. Finally, in Figure 17, we show a combined plot of the growth rate $G_{T}$ for the Equal Receptor and Adaptive receptor strategies against the corresponding MI MI(T), both as a function of an increasing γ factor. The simulation parameters used to obtain these results are the same as for the results shown in Figure 13.

The noise factor γ is defined as the ratio between the variance of a Gaussian distribution and the variance of the Binomial distribution defined in (2), respectively. This Gaussian distribution is utilized in place of the Binomial in (2) to obtain the results in Figure 14, Figure 15 and Figure 16 as a function of a varying receptor noise. This is expressed as a Gaussian with the same average as the corresponding Binomial, and a variance equal to the variance of the corresponding Binomial multiplied by the noise factor γ, as

(19) $$A_{R}^{*}∼N\left(\frac{A_{\mathrm{tot}}}{2}p_{\mathrm{bind},A_{R}},\left(\frac{A_{\mathrm{tot}}}{2}\right)p_{\mathrm{bind},A_{R}}\left(1-p_{\mathrm{bind},A_{R}}\right)\gamma \right),$$

where $A_{\mathrm{tot}}$ and $p_{\mathrm{bind},A_{R}}$ are defined in (2). We utilize similar Gaussian distributions with analogous definitions for $A_{L}^{*}$, $B_{R}^{*}$, and $B_{L}^{*}$, respectively.

## 6. Discussion

The cell division and cell density distributions plotted in Figure 4 and Figure 5 show how the cell strategies are responding to stress and the static substrate distribution in the environment. Considering that the cells with the equal receptor allocation strategy have a lower growth rate, as shown in Figure 13, the higher density of cells in the interval of two concentration peaks is likely a necessity in that cells in this region would be close to either peak in order to stay alive. In contrast, the cells in the adaptive receptor allocation strategy are clustering around the two highest peaks of the ${\left[A\right]}_{i}$ and ${\left[B\right]}_{i}$ distributions. Considering that these cells have a comparatively higher growth rate, this can be interpreted as the cells’ ability to stay in higher A or B concentration regions for longer.

When a large number of cells have a higher division rate in a particular region of the environment that region’s average effect on the cells’ state will result in a higher density. The cell density in Figure 6, for the equal receptor allocation strategy, is larger near the origin. Considering that the cell’s divide density location in Figure 4 is centered around the middle of the two concentration masses, the larger number of cell divisions in this region could reveal that the majority of the distribution of the cells exist in a state having an equal and relatively low, compared to the adaptive allocation strategy, internal storage $A_{\mathrm{in}}$ and $B_{\mathrm{in}}$. This is also consistent with Figure 13, where the equal receptor allocation strategy has a lower growth rate compared to the adaptive receptor allocation strategy. Conversely, in Figure 7, the adaptive receptor allocation strategy has a spread-out cell density with a larger portion contained in the area corresponding to less than 50 $A_{\mathrm{in}}$ and 50 $B_{\mathrm{in}}$. This can be interpreted as this strategy allowing for a larger internal storage of $A_{\mathrm{in}}$ and $B_{\mathrm{in}}$ during the simulation.

Both receptor allocation strategies have a sharp cutoff in the $A(\bar{x})$ and $B(\bar{x})$ when $A_{\mathrm{in}}$ or $B_{\mathrm{in}}$ are less than 10 (Figure 8, Figure 9, Figure 10 and Figure 11), which is likely due to cell death in the simulation. In this region of the heat map plots, there is a non-zero cell density, albeit very low. This can be interpreted as the cells’ chance of survival being greatly reduced for these low values of internal storage.

The equal receptor allocation strategy has a relatively uniform density of internal storage $A(\bar{x})$ and $B(\bar{x})$ along a strip between $B_{\mathrm{in}}=\left[10,25\right]$ and $A_{\mathrm{in}}=\left[10,25\right]$, as shown in Figure 8 and Figure 9. The adaptive receptor allocation strategy in this same strip is not as uniform, while a high density in $A(\bar{x})$ and $B(\bar{x})$ occurs when $B_{\mathrm{in}}=60$ and $A_{\mathrm{in}}=60$, respectively.

The results shown in Figures Figure 12 and Figure 13 clearly demonstrate that, *for every value of cell stress imposed in the simulation, the adaptive receptor allocation strategy results in a **higher growth rate and correspondingly lower average** MI(T) than the equal receptor allocation strategy*, where these differences increase as the cell stress increases. In addition, as the cell stress increases, both allocation strategies result in not only a lower growth rate, which is expected, but also a higher average MI(T). This might be explained by how cells survive with respect to their strategy [33]. With higher stress, cells with a higher “capacity” to receive information (either because of their locations or their receptor allocations) from the environment have a higher probability to be naturally selected over others. This selection seems more pronounced for the equal receptor allocation and more subtle for the adaptive strategy, where cells tend to adapt their “capacity” to the more stringent constraint and increase their fitness.

In Figure 14, the initial rise in input entropy for low noise, as the noise increases, is due to a higher mobility of the cells to sample more diverse environmental locations, and a higher range of different $A_{\mathrm{in}}$ and $B_{\mathrm{in}}$ values. The corresponding growth rate in Figure 15 and mutual information in Figure 16 for the equal receptor allocation seem to reach optimal values (maximum growth with minimum mutual information) before plunging when the noise increases further. The same does not happen for the adaptive receptor strategy. The input entropy then stabilizes for both strategies after the noise factor γ reaches value 1. For higher noise, the subtle variations in the conditional entropy seem to dominate the variations in MI(T), shown in Figure 16, which is therefore more stable, as the growth rate in Figure 15. As the noise increases, MI(T) and the growth rate decrease for both strategies, as expected. Nevertheless, for all the noise values, the adaptive receptor allocation results in higher growth rates, achieved with a lower mutual information for all but in the aforementioned optimal noise region for the equal allocation.

The combined plot in Figure 17 offers the opportunity of a direct comparison with the work presented in [20]-(Figures 1c, 2b, 3b and 4b). In particular, while our model does not allow for an independent selection of the mutual information between organisms and environment during simulation, it is clear that the definition of “semantic information” from [20] is here not directly applicable since the degradation of the information channel operated by an increased noise is in our case countered by an optimization of the channel by each individual organism in the simulated population. This cannot be represented by a single average mutual information, and underscoring the need for a metric to measure the emergence of “subjective information”, i.e., mutual information between environment and organism that varies across individuals in the same population.

### Measuring the Emergence of Subjective Information

**Figure 18:**
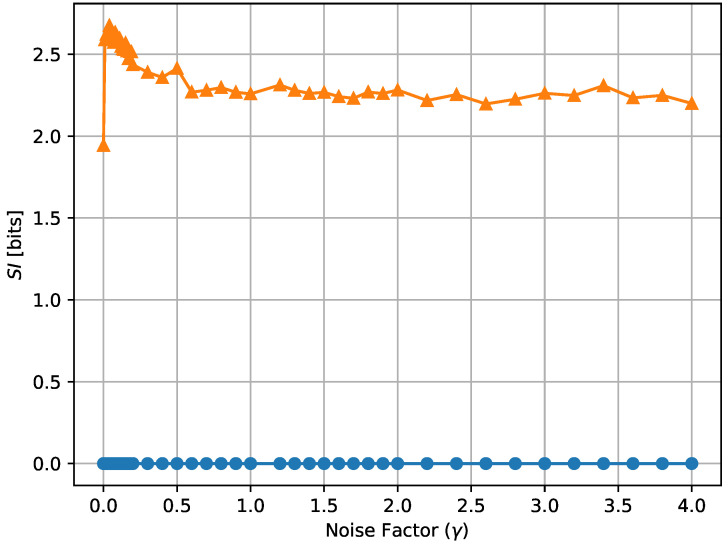
Subjective information of the equal receptor and adaptive receptor strategies as the noise factor (γ) is increased. The simulation parameters used to obtain these results are the same as for the results shown in Figure 13.

**Figure 19:**
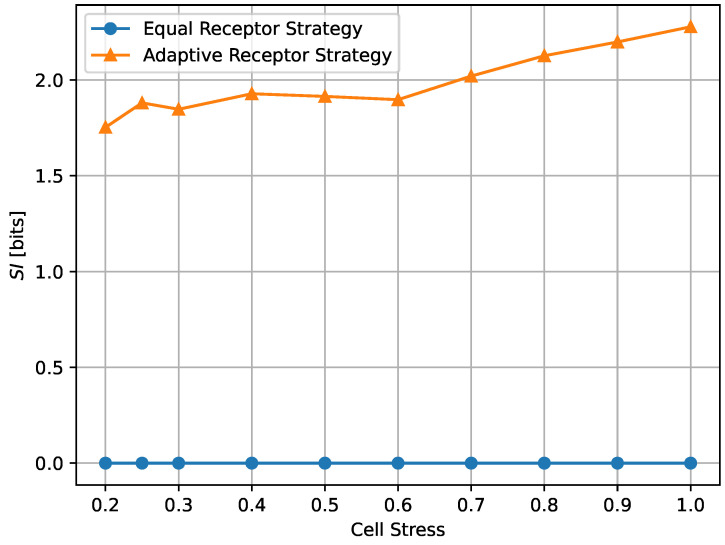
Subjective information of the equal receptor and adaptive receptor strategies as the cell stress factor is increased. The simulation parameters used to obtain these results are the same as for the results shown in Figure 13.

Given the results detailed above, we proposed to measure the emergence of “subjective information” (SI) over a time *T* by taking the average over time of the standard deviation of the MI of each individual cell $MI_{cell}$ over the population, as

(20) $$SI=E_{T}\left[\sigma_{\mathrm{Total}\#\mathrm{of}\mathrm{cells}}\left(MI_{cell}\right)\right],$$

where σ(.) denotes the standard deviation operator. According to this definition, the equal receptor allocation strategy has by definition SI equal to 0, as demonstrated also empirically in Figure 18 and Figure 19, while the adaptive allocation strategy shows a positive SI in all considered situations. The proposed measure is relatively stable as a function of the noise, as shown in Figure 18, but, in the case of the adaptive receptor allocation strategy, it has an overall tendency to increase with cell stress. We reserve further study of “subjective information” measurements to future work.

## 7. Conclusions

In this paper, we introduced a simulation-based scenario to analyze the emergence of a form of “subjective information” and its correlation with the survival of a biological organism. In particular, we defined an abstract mathematical model able to capture the parameters and behaviors of a population of single-celled organisms while they move through chemotaxis to absorb two essential nutrients from the environment. This model is then translated into a computational state machine, which is then utilized in a simulation framework constructed specifically to characterize and measure the emergence of the aforementioned information. For this, two different strategies adopted by these cells are considered, namely, based either on maximizing information on the two essential substrates or, alternatively, reducing this information by focusing on what is more important for survival. Simulation results based on these two strategies for different parameters related to cell’s survival stress are compared in terms of information efficiency of the cells Vs growth rate. The obtained results clearly reveal that the strategy that maximizes information efficiency results in a lower growth rate with respect to the strategy that has lower information efficiency but optimizes the information channel of each individual to better focus on the pieces of information from the environment that are more important for survival, i.e., the “subjective information”.

A more robust model and in vivo experiments will be required to better define and quantify “subjective information” of a living system. With this analysis, this new information concept may identify an important aspect of biological systems that can be used in tandem with other information theoretic principles studied in prior literature. 

## Data Availability

All data presented in this paper, together with the corresponding source code, can be found at https://github.com/tbarke/BioSimulator (accessed on 5 January 2022).
